# Understanding the Relationship between the Molecular Structure and Physicochemical Properties of Soft Rice Starch

**DOI:** 10.3390/foods12193611

**Published:** 2023-09-28

**Authors:** Congnan Zhang, Wei Xue, Ting Li, Li Wang

**Affiliations:** 1 Key Laboratory of Carbohydrate Chemistry and Biotechnology Ministry of Education, School of Food Science and Technology, Jiangnan University, Lihu Road 1800, Wuxi 214122, China8202302001@jiangnan.edu.cn (T.L.); 2 National Engineering Research Center for Cereal Fermentation and Food Biomanufacturing, Jiangnan University, Lihu Road 1800, Wuxi 214122, China; 3 Jiangsu Provincial Engineering Research Center for Bioactive Product Processing, Jiangnan University, Lihu Road 1800, Wuxi 214122, China

**Keywords:** soft rice starch, molecular structures, physicochemical properties, chain-length distributions

## Abstract

The relationship between the molecular structure and physicochemical properties of soft rice starch (SRS) has been investigated in this research. The amylose content of SRS ranged from 10.76% to 11.85%, classified as the very low amylose type. Compared to waxy and japonica rice starch, the largest amount of small starch granules and the highest viscosity were shown in the SRS. The results of X-ray diffraction and Fourier transform infrared proved that the SRS depicted a typical A-type pattern with a low short-range ordered structure. Additionally, SRS had a great deal of A and B_1_ chains. Molecular weights and density of starch from soft rice were lower than those from waxy rice but higher than those from japonica rice. Furthermore, SRS possessed a higher amount of resistant starch. Correlation analysis indicated that the amylose content and the chain-length distributions of amylopectin play a major role in influencing the molecular structure and physicochemical properties of rice starch. In conclusion, the low amylose content, highest viscosity, and other excellent properties of soft rice starch make it have bright application prospects in instant rice and rice cakes.

## 1. Introduction

Rice is the most common cereal and predominant principal food crop worldwide, which provides daily nutrition and calories for the majority of people [1]. Along with the growing demand for rice products with high quality, it is important for breeders to improve the rice grain quality [2]. In recent years, Chinese rice breeders have successfully developed a series of new soft rice varieties with excellent performance. These soft rice showed excellent eating and cooking quality with low amylose content ranging from 8% to 12% [3]. The cooked soft rice is soft, sweet, non-sticky, and has good palatability after cooling compared to waxy rice and japonica rice. The principal constituent of rice grain is starch (>80%, dry weight basis), of which physicochemical properties and structures pose a great influence on the edible quality of cooked rice [4]. 

Rice starch is mainly composed of two types of polymers, linear amylose and highly branched amylopectin. The amylose content of rice starch ranges from 0 to 35%. According to the amylose content, rice starch can be classified as waxy (<2%), very low (5–12%), low (12–20%), intermediate (20–25%), and high (>25%) amylose starch [5]. The amylose content was the lowest in waxy rice and highest in indica rice. Starch composition and structure are closely linked with the physicochemical and cooking properties of rice. Starch properties are determined by a large number of factors, such as amylose content, degree of crystallinity, starch granule size, gelatinization behaviors, and amylopectin fine structure. Amylose content was reported as the principal factor influencing the physicochemical properties of rice starch. The pasting and rheological properties, which are closely related to thermal processing, rely greatly on amylose content and granular structure [6]. The degree of crystallinity and gelatinization temperature have been proposed to be related to the retrogradation properties of rice starch [7]. Also, amylose content and the short-range ordered structure have an impact on the swelling power and water solubility index of rice starches [8]. In addition, the digestion rate of indica rice starches is influenced collaboratively by amylose content and molecular short-range order. The differences in granule size and shape and starch granule size distribution suggested a remarkable influence on the functionalities of rice starch [9]. However, these researches are mainly aimed at the waxy, japonica, or indica rice. In the last several years, the breeding of soft rice has developed rapidly, and some soft rice varieties with good edible quality have been launched successfully. Most rice breeders have paid great attention to these new varieties, such as Nanjing and Suxiangjing soft rice. The quality of these new varieties is equal to high-quality rice from northeast China and Japan. To our knowledge, many studies are mostly concentrated on breeding and genetic projects, and there is limited information to investigate the physicochemical characteristics and molecular structure of starch from soft rice [10,11]. The research in this area is of great significance for rice breeders in cultivating high-quality rice varieties and making good use of rice starch.

This paper is aimed to study the physicochemical characteristics and molecular structure of soft rice starch (SRS). Its morphology, granule size, thermal properties, pasting properties, crystal structure, short-range ordered structure, cooking properties, in vitro digestibility, molecular weight distribution, and amylopectin chain-length distribution were studied. The correlation between physicochemical and molecular properties was analyzed. These results will provide the necessary knowledge for their potential application in the planting and food industries.

## 2. Materials and Methods

### 2.1. Materials

Seven paddy cultivars, including two waxy rice (99-25 and Wuyunuo 481—abbreviated as WYN), three soft rice (Nanjing 46—NJ 46, Nanjing 9108—NJ 9108, and Suxiangjing 100—SXJ 100), and two japonica rice (Daohuaxiang 2—DHX and Akitakomati—Aki), were selected. The waxy and soft rice samples were planted and harvested in Jiangsu province, China. These rice varieties selected above were typical and widely cultivated in China. Total Starch Assay Kit, porcine pancreatic α-amylase, amyloglucosidase, and D-glucose assay kit were purchased from Megazyme Co., Ltd. (Wicklow, Ireland). 

### 2.2. Preparation of Rice Flour

The moisture content of paddy samples was controlled at 12~15%. All cleaned paddy samples were dehulled using a paddy huller (THU35C, Satake, Japan) to obtain brown rice and milled by a vertical rice whitening machine (TM05C, Satake, Japan) to get milled rice. The milled rice was ground in the frozen grinding machine and passed through a 100-mesh sieve to collect the rice flour. After completing the above processes, the rice flour was stored in a glass desiccator for subsequent analysis. All the experiments were performed in triplicate unless otherwise specified.

### 2.3. Starch Extraction

The starch was extracted from rice flour by the alkaline steeping method with slight modifications [7]. Rice flour (50 g) was dispersed in a 0.2% NaOH solution under stirring at 25 °C for 30 min before being left at room temperature for 12 h. After that, the dispersion was filtrated through 200 mesh sieves. Then, the filtrate was washed using distilled water and centrifuged at 4000× g for 10 min; the supernatant was removed from the top yellow protein layer. The starch pellet was resuspended in distilled water and the above procedures were repeated seven times until the yellow layer was completely removed. Finally, the white starch sediment was dispersed in distilled water (250 mL) and then neutralized to pH 7.0 using 0.1 M HCl solution. Then, the resulting samples were centrifuged at 4000× *g* for 10 min to wash the sediment. The washing step was repeated more than six times to ensure that the protein was removed successfully. The extracted rice starch was dried at 40 °C in an oven and collected with a 100-mesh sieve for subsequent analysis.

### 2.4. Determination of Amylose Content

The determination of amylose content usually used the iodine reagent method (GBT15683-2008). Three grams (dry basis) of rice starch was defatted with 85% (*v*/*v*) methanol and was dried at 40 °C; 1 mL of 95% (*v*/*v*) ethanol was added to 100 mg rice starch sample in a conical flask, then 9 mL of 1.0 mol/L NaOH was added and mixed slightly. The suspension of the flour was incubated with boiling water for 10 min and cooled to room temperature with ice water, then transferred to a 100 mL volumetric flask and fully mixed. Then, 5 mL of solution was transferred to another 100 mL volumetric flask with a pipette and 1 mL of 1.0 mol/L acetic acid and 2 mL of iodine reagent were added in sequence. Then, 1.5 mL solution was transferred to a cuvette and the absorbance was determined by a spectrophotometer (Mepda Instruments Co., Ltd., Shanghai, China) at 720 nm after 10 min. 

### 2.5. Morphology and Particle Size Distribution of Starch Granules

The rice starch granule morphology was observed with scanning electron microscopy (Quanta-200; FEI Ltd., Hillsboro, USA). Starch samples were fixed on the aluminum sheet with double-sided tapes and coated vertically gold. The accelerating voltage and magnifications were 5.0 kV and 2400×, respectively [8].

The particle size distribution of rice starch samples was measured using a laser diffraction particle size analyzer (BT-9300S, BETTER, Shanghai, China). The starch samples (200 mg) were suspended in distilled water (2 mL) and stirred fully at 1500 rpm. Then, the instrument program is selected to determine granule size ranging from 0.1 to 2000 μm. The volume size distribution of starch granules was determined and recorded. 

### 2.6. Short-Range Molecular Order

The short-range molecular order of rice starch was measured by the IS10 27 Fourier transform infrared spectroscopy (Nicolet, Madison, WI, USA). The ratio of starch samples and KBr powder was mixed in a ratio of 1:150 (*w*/*w*) and then pressed into 2 mm pellets. The spectra scanned times, wavenumber range, and resolution were 64, 4000 to 400 cm^−1^, and 4 cm^−1^, respectively. The spectra at 1200–800 cm^−1^ were analyzed with baseline correction before deconvolution by OMNIC 8.2. The width and enhancement factor were 38 cm^−1^ and 1.9, respectively.

### 2.7. Starch Crystalline Structure

The relative crystallinity and structure of the crystalline region of rice starch were determined by an X-ray diffractometer (XRD) (D2 PHASER, Bruker, Germany) operated at 40 kV and 40 mA Cu-Kα radiation. The diffraction angle (2θ), scanning speed, and step width were 5° to 40°, 2°/min, and 0.02°, respectively. Before measurements, all starch samples were placed in a glass desiccator, where a constant humidity atmosphere was maintained by the saturated NaCl solution at room temperature for 24 h. The relative crystallinity of rice starch was calculated by JADE 6.5 software.

### 2.8. Determination of Thermal Properties

Differential scanning calorimetry (DSC3, Mettler, Switzerland) was used to measure the thermal properties of rice starch samples. Three milligrams (dry basis) of starch samples and 9.0 μL of distilled water were added to an aluminum pan. The empty pan was considered as the reference, and the pans were sealed and equilibrated for 12 h at 4 °C prior to the measurement. The heating range and rate were 25 °C to 100 °C and 10 °C/min, respectively. The gelatinization temperature was calculated using data recording software, including onset (To), peak (Tp), and conclusion (Tc) temperatures, the gelatinization range (Tc-To), along with the gelatinization enthalpy (ΔH). 

### 2.9. Pasting Properties, Textural Properties, and the Clarity of Starch Pastes Determination

The pasting profiles of rice starch samples were determined by using Rapid Visco-Analyser (RVA 4500, Perten, Australia). About 3.0 g (dry basis) of starch samples and 25 mL of distilled water were added to the canister. The starch suspension after being stirred underwent a heating and cooling process programmed by the instrument. The measurement procedure is shown as follows. Firstly, the starch suspension started at 50 °C for 1 min before increasing to 95 °C with a heating rate of 12 °C/min. Secondly, equilibrated at 50 °C for 1 min. Finally, cooled to 50 °C with a heating rate of 12 °C/min and maintained for 4 min at 50 °C. Meanwhile, the speed of rotation was 960 rpm for the first 10 s, then increased to 160 rpm and maintained for rest time during measurement. 

The textural properties were assessed using a texture analyzer (TA.XTC-20, Shanghai Baosheng, Shanghai, China). Five grams of starch sample was suspended in 40 mL water and stirred at 300 rpm for 20 min. The suspensions were heated in a water bath at 95 °C for 20 min with continuous stirring to ensure complete gelatinization. Then the pastes obtained were immediately transferred into cups (40 mm diameter, 10 mm height) with a cover and stored at 4 °C for 12 h. The samples were tested on the sample table of the texture analyzer, and the test conditions were set as follows: the P36 probe was selected, the test speed was 1 mm/s, and the pre-speed and post-speed were both set at 2.0 mm/s. The deformation level was set at 20% of the original sample height in order to avoid the complete destruction of starch gel in the first compression.

The starch sample (100 mg) and distilled water (10 mL) were weighed into screwcap tubes and mixed evenly. The tube was heated in a boiling water bath for 30 min and shaken every 5 min. After cooling to room temperature, the percentage of transmittance (% T) at 650 nm was measured by spectrophotometer (UV-3200, Mapada Instruments Inc., Shanghai, China) against distilled water blank.

### 2.10. Starch Swelling Power (SP) and Water Solubility Index (WSI)

The SP and WSI were measured by Wei’s [12] method with slight modifications. Briefly, 0.5 g starch samples were weighted gently into a 50 mL centrifugal tube, and then 10 mL distilled water was added. Then, the starch suspension was fully mixed and incubated at 95 °C for 30 min with continuous shaking in a water bath. After that, the resulting suspension was cooled to 25 °C and centrifuged at 10,000× *g* for 15 min. The supernatant was transferred to an aluminum box with a pipette and dried to a constant weight (W_1_) in an air oven at 105 °C. The sediment that was attached to the wall of the centrifugal tube was weighted (Ws). The SP and WSI were calculated by the following equations.

(1) $$\mathrm{WSI}=\frac{W_{1}}{0.5}\times 100\%\;\;$$

(2) $$\mathrm{SP}=\frac{W_{S}}{0.5\times \left(100\%-\mathrm{WSI}\right)}\;\;$$

### 2.11. Determination of Molecular Weight

The molecular weight distribution of the rice starch was determined by a size exclusion chromatography-multi-angle light scattering-refractive index detector (SEC-MALLS-RID) (Wyatt Technologies, Santa Barbara, CA, USA). Starch samples (50 mg) were dissolved in dimethyl sulfoxide (DMSO) solution with 50 mM LiBr. The starch dispersion was heated at 80 °C with continuous shaking (100 rpm) overnight. The solution was passed through a 0.22 μm organic membrane filter before being injected into the SEC-MALLS-RID system with the He-Ne laser source (658 nm). Three SEC columns (Styragel guard column 4.6 × 30 mm, Styragel HMW 2 DMF 7.8 mm × 300 mm, Styragel HMW 6E DMF 7.8 mm × 300 mm) (Waters, Milford, CT, USA) were used to analyze the molecular weight and the column temperature was maintained at 60 °C. DMSO solution containing 50 mmol/L LiBr was the mobile phase, and the flow velocity was 0.6 mL/min. The dn/dc was 0.04 mL/g to calculate the data. Number average molecular weights (M_n_), polydispersity index (PDI = Mw/Mn), z-average radius of gyration (Rz), and molecular density (*ρ* = Mw/Rz^3^) of rice starches were obtained from Astra software version 5.3.4.

### 2.12. Branch Chain-Length Distributions of Amylopectin

The chain-length distributions of amylopectin were determined by the SEC coupled with a refractive index (RI) detector. Starch samples and DMSO (50 mg: 10 mL) were mixed and heated at 90 °C with shaking at 160 rpm overnight. Thereafter, 1 mL of sample was transferred and mixed with 6 mL of absolute ethanol. Then, the suspension was separated by centrifugation (5000× *g*, 20 min) to precipitate the starch granules, and the starch sediment was redissolved in the boiling water for 10 min and then cooled to 50 °C. Sodium acetate (40 μL, 50 mM, pH 3.5) and pullulanase (10 μL, Megazyme, Ireland) were added to the starch solution. Then, the starch solution was debranched at 50 °C for 48 h. The reaction was stopped by boiling for 10 min. Then, the solution (20 μL) was passed through a 0.22 μm membrane filter and injected into the SEC-RID system. Three SEC columns (Shodex OHpak SB-G 6B, SB-804 HQ, and 802.5 HQ) (Showa Denko, Tokyo, Japan) controlled at 60 °C. The system was calibrated with Pullulan P-82 (Showa Denko, Tokyo, Japan). The mobile phase was ammonium acetate (50 mM, pH 5.2) and the flow rate was 1.0 mL/min.

### 2.13. In Vitro Starch Digestibility

The in vitro starch digestibility of rice starch was determined by the method of Englyst, Kingman, and Cummings with minor modifications as follows [13]. One hundred milligrams (M_0_, dry basis) of rice starch were incubated in a 5 mL solution composed of porcine pancreatic α-amylase (290 U/mL) and amyloglucosidase (50 U/mL) in 0.1 M sodium acetate (pH 5.2) at 37 °C with continuous shaking (160 rpm) for 200 min. Aliquots (100 μL) were taken and added to absolute ethanol (900 μL) at 0, 5, 10, 15, 20, 40, 60, 80, 100, 120, 160, and 200 min to stop the enzymatic reaction. The mixture was centrifuged at 4500× *g* for 10 min, and the glucose content was determined in the supernatant by the GOPOD kit (Megazyme, Ireland). The rapidly digestible starch (RDS) content, slowly digestible starch (SDS) content, and resistant starch (RS) content were calculated by the following equation. G_20_ and G_120_ represented the glucose content that was digested at 20 and 120 min, respectively.

(3) $$\mathrm{RDS}\left(\%\right)=\frac{G_{20}\times 0.9}{M_{0}}\times 100$$

(4) $$\mathrm{SDS}\left(\%\right)=\frac{(G_{120}-G_{20})\times 0.9}{M_{0}}$$

(5) $$\mathrm{RS}\left(\%\right)=100-\mathrm{RDS}-\mathrm{SDS}$$

### 2.14. Statistical Analysis

Data in all tables were expressed using “means ± standard deviation”. The group means were compared using analysis of variance with Duncan’s post hoc test, and all statistical analyses were performed using SPSS 21.0 and *p*-values < 0.05 were considered as statistically significant.

## 3. Results and Discussion

### 3.1. Amylose Content, Morphology, and Particle Size Distribution of Starches

The amylose content of different rice starches is presented in Table 1. There was an obvious difference in the amylose content among various rice starches. The amylose content of rice starch varied from 0% to 21.60% in different rice varieties. Waxy rice starch (WRS) presented the lowest amylose content, while japonica rice starch (JRS) presented the highest amylose content. The amylose content of SRS ranged from 10.76% to 11.85%, classified as a very low amylose (5–12%) type. In general, the amylose content plays an important role in the qualities of cooked rice, especially the eating and tasting qualities. The amylose content of traditional rice varieties varied from 0.1% to 28.7% [6,7,14]. The amylose content was also influenced by the different botanical sources and growing environments, such as climate, fertilization, topographical, soil, and planting parameters, during the development of rice grain [6,15].

**Figure 1:**
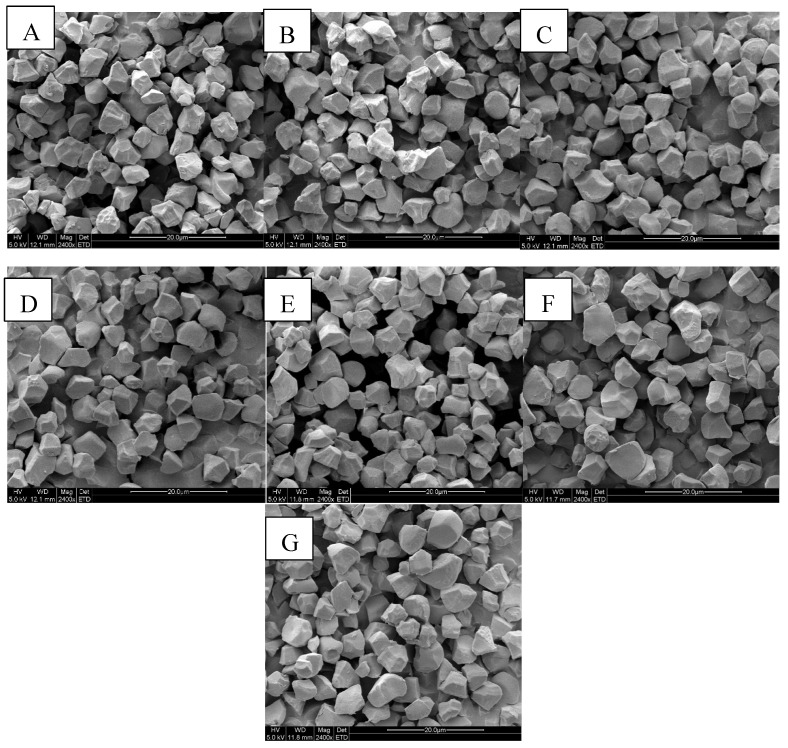
Scanning electron micrographs (SEMs) of starches separated from waxy, soft, and japonica rice cultivars: 99-25 (**A**), WYN (**B**), NJ 9108 (**C**), SXJ 100 (**D**), NJ 46 (**E**), DHX (**F**), and Aki (**G**).

The starch granules morphology was determined using scanning electron microscopy, which is shown in Figure 1. The similarly polyhedral and irregular sharp angles and edges were exhibited in these starch granules. These results were consistent with previous studies [7,16]. The surface morphology of WRS was rougher than that of others. There was no obvious difference in granule size. Wani reported that starch granule morphology was affected by rice genotypes, isolation methods, climatic changes, cultivar technology, and starch biosynthesis [5].

The starch granules from various rice cultivars exhibited a remarkable difference in particle size distribution of starch (Table 1). The small starch granule (<2 μm) proportion of these starches ranged from 16.80% to 21.55%. The small starch granule proportion of SRS varied from 21.12% to 21.55%, which was higher than the waxy and japonica rice starch. The results might be attributed to the difference in genotype, growing conditions, and fertilization. Waduge et al. [17] reported that large starch granules first come out in the filling period and then decompose into medium-sized and small starch granules. Therefore, the primary small particles and subordinate large particles coexisted in final starches. In addition, the granule distribution is also affected by cultivation conditions, such as nitrogen content and temperature in the filling duration [18].

### 3.2. Crystalline Structure of Starch

**Figure 2:**
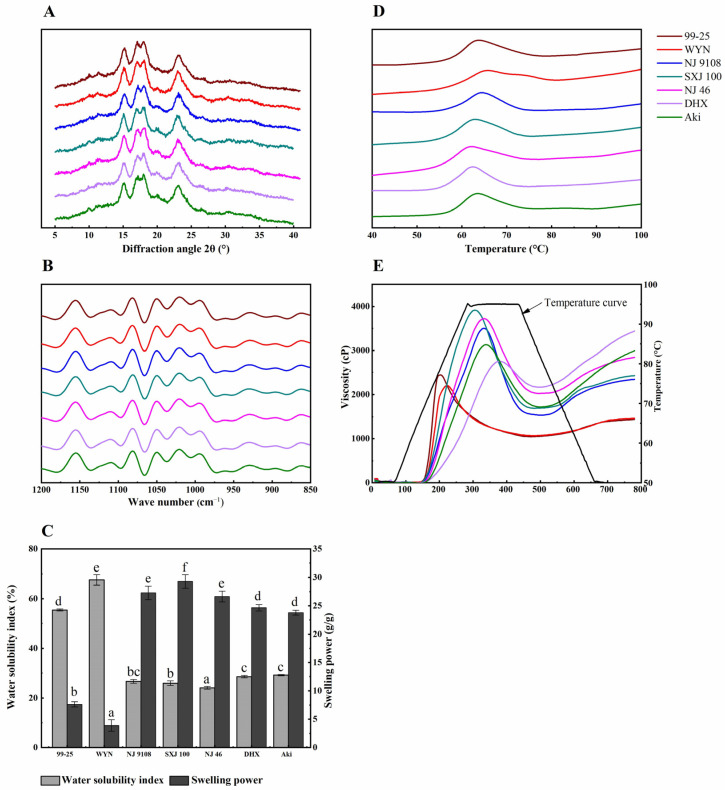
XRD patterns (**A**), FTIR spectra (**B**), swelling powder and water solubility index (**C**), thermal properties (**D**), and pasting properties (**E**) of starches separated from waxy, soft, and japonica rice cultivars. Different letters in the same indicators show significant differences (*p* < 0.05).

The different rice starches showed similar XRD patterns, as shown in Figure 2A. All rice starches depicted a classical A-type pattern with the main angle diffraction peaks at 15.3°, 17.1°, 18.2°, and 23.5°, which was in accordance with the patterns of the previous study [19]. The relative crystallinity of various rice starches is presented in Table 1. The relative crystallinity varied from 22.05% (DHX) to 31.15% (WYN) among the given rice cultivates, which was influenced by the starch granule size, especially the small granule content. The small starch granules resulted in lower relative crystallinity [20]. Differences in the relative crystallinity of starch from a variety of rice cultivars could be ascribed to the amylose content and fine structure of amylopectin [15]. The relative crystallinity of SRS (26.15~27.55%) was lower than WRS while was higher than JRS. The results might be attributed to the low amylose content and less long-chain of amylopectin in SRS. The highest relative crystallinity was exhibited in the starches of the WRS among the different rice varieties. It was found that relative crystallinity was negatively related to the amylose content, which agrees well with the previous report [21]. 

The FT-IR spectra in the range of 1200–800 cm^−1^ for different cultivars of rice starch were similar (Figure 2B). FT-IR spectra are extremely sensitive to the short-range ordered structure, which is widely used to identify the double-helix content of the external region of starch. The FTIR absorbance band at 1022 cm^−1^ is linked with the amorphous region that is affected by stretching modes in starch, and the band at 1047 cm^−1^ is associated with the order/crystalline region in the starch granule. The ratio of 1047/1022 cm^−1^ can be used to characterize the short-range order degree in starch, and the ratio of 1022/995 cm^−1^ is the ratio of amorphous to ordered starch structure [22]. The ratio of 1047/1022 cm^−1^ and 1022/995 cm^−1^ varied among the different rice starches (Table 1). The ratios of 1047/1022 cm^−1^ varied from 0.842 to 0.907 in different rice starches. The highest and lowest values were found in 99-25 and NJ 46, separately. The ratios of 1047/1022 cm^−1^ of SRS were ranged from 0.842 to 0.862. The results illustrated that the short-range order degree was lower than that of WRS. However, no significant difference between SRS and JRS was observed. The ratios of 1022/995 cm^−1^ were the highest in SRS (1.345) and the lowest in WRS (1.200). The short-range ordered structure was principally influenced by the crystalline form and starch granule size [23].

### 3.3. Thermal Properties

The thermal curve of different rice cultivar starches was similar (Figure 2D). The values of gelatinization temperature (To represents onset temperature, Tp reflects peak temperature, and Tc is conclusion temperature) and the value of temperature enthalpy presented a significant difference in all rice starches (Table 2). NJ 46 exhibited the lowest To (55.11 °C) and Tp (61.75 °C), while NJ 9108 presented the highest To (57.55 °C) and Tp (64.37 °C). WYN showed the highest Tc (79.53 °C), ΔH (11.83 J/g), and Tc-To (23.65 °C), whereas DHX exhibited the lowest Tc (69.75 °C), ΔH (8.88 J/g), and Tc-To (13.70 °C). The ΔH of SRS ranged from 10.00 to 11.57 J/g, which was higher than JRS while lower than WRS. The result might be ascribed to the low amylose content and the fine structure of amylopectin. Compared to the SRS, WRS had higher Tc, ΔH, and Tc-To, which agrees well with the result of Wang et al. [11]. The differences in gelatinization temperature from rice starches could be ascribed to the amylose and amylopectin content, granule size, amylose, as well as amylopectin fine structure, such as molecular weight and chain-length distributions [23]. Gelatinization enthalpy (ΔH) is defined as a symbol of the quantity and quality of starch crystallinity, which reflected the losses of the double-helix and crystalline structures [24]. The high short-range order and high relative crystallinity could lead to the high ΔH in this study, as shown by FT-IR and XRD. These results agreed well with the study of Chiotelli [25], who found that ΔH was positively linked to the short-range ordered structures and relative crystallinity.

### 3.4. SP and WSI of Starch

The crystal structure of the starch granules is destroyed due to the hydrogen bond fracture when starch interacts with excess water during heating. Furthermore, hydrogen bonds are formed between the water molecules and exposed starch, leading to starch swelling and increased solubility [5]. The SP and WSI reflected the interaction between the water molecules and starch chains in crystalline and amorphous areas [26]. The SP and WSI of different rice starches are depicted in Table 2. The SP ranged from 3.89 to 29.30 g∙g^−1^ and the WSI ranged from 24.09 to 67.57% in various rice starches. WYN exhibited the lowest SP and highest WSI. The highest SP was observed in the SRS and higher than 26 g∙g^−1^. The WSI of SRS was about 25%, which was lower than JRS. The difference in SP was primarily due to the amylose content, and the double-helix structure of amylose could restrain starch swelling and retain the swollen granules [27]. Moreover, the fine structure, the ratio of amylose and amylopectin, amorphous, and crystalline structure could also affect the SP of starch. WSI was influenced by the soluble molecules such as amylose and the proportion of short-chain in amylopectin [28,29].

### 3.5. Pasting and Textural Properties of Starch

The pasting properties among the analyzed rice starches differed significantly (Table 2), and the viscosity curves are shown in Figure 2E. Peak viscosity (PV) reflects the capacity to bind water of granules and affect the texture of the final product quality. PV of rice starch was influenced by amylose content. The PV of samples was varied from 2185 to 3962 cP. Furthermore, SXJ 100 showed the highest PV and WYN presented the lowest PV. In addition, the PV of starch from soft rice was higher than that of waxy rice and japonica rice. In our study, WRS presented the lowest PV. Similar results had been reported on rice starches in a previous study [30]. The SRS showed the highest PV (3614-3962 cp), which may be related to the cluster structure of amylopectin and the highest percentage of the small starch granule. When the viscosity of the starch paste rises, the corresponding temperature is currently called the gelatinization temperature (P_Temp_). P_Temp_ varied from 67.4 (99-25) to 71.8 °C (DHX). The WRS presented the lowest P_Temp_ and PV. The result was in line with the study of Wang [11]. P_Temp_ of SRS ranged from 69.8 to 70.2 °C, which was lower than P_Temp_ of JRS. Sang et al. [31] demonstrated that amylose–lipids complexes could inhibit starch granules swelling and lead to a lower PV at a higher P_Temp._ This was not enough to account for the current results. The results showed that the PV and P_Temp_ were also affected by other important factors besides the swelling powder. Hot paste viscosity (HPV) ranged from 1037 to 2140 cP among different rice starches with the lowest in 99-25 and the highest in DHX, and SRS ranged from 1544 to 2006 cP. Cold paste viscosity (CPV) varied from 1434 to 3438 cP with the lowest for WYN and the highest for DHX. The CPV of SRS was lower than JRS while higher than WRS, ranging from 2361 to 2818 cP. The increase in CPV is linked to the rearrangement of amylose and amylopectin during the cooling process [32]. The higher CPV may be ascribed to the fact that the amylose inhibits granule swelling. Breakdown viscosity (BD) can be used to measure the heat and shear resistance of paste and evaluate the vulnerability of starch granules, which indicates stability in the cooking process. Adebowale et al. [33] reported that the higher BD represented the worse heat-resistant ability of the starch. BD ranged from 620 to 2265 cP, corresponding to DHX and SXJ 100, respectively. The SRS had the highest BD that varied from 1736 to 2265 cP, and the changes in BD could be attributed to the different hardness of the swollen starch granules. Finally, the setback viscosity (SB) reflects the retrogradation tendency of starch paste, which can be used to indicate the difficulty of starch retrogradation. SB ranged from 379 to 1300 cP corresponding to WYN and Aki starches, respectively. The SB of soft rice starch varied from 726 to 818 cP, which was higher than starch from waxy rice but lower than that of japonica rice. These results indicated that SB was affected by the amylose content. Amylose fraction is more likely to retrograde than amylopectin due to the amylose linear structure, and long-chain structure contributes to the formation of hydrogen bonds and the firmness of the gel [34]. The time that reaches the PV is called peak time (P_time_) during the RVA heating period. The shortest P_time_ was found in 99-25 (3.36 min) while the longest P_time_ was in DHX (6.20 min). In this study, the SRS achieved the highest PV and BD among different rice starches, and the HPV, CPV, SB, P_time_, and P_temp_ were between WRS and JRS. 

The textural properties of different rice cultivars starches are given in Table 2. The hardness and gumminess of starch from soft rice were lower than those from japonica rice but higher than those from waxy rice. Amylose content has been reported to be positively related to the gel hardness [6]. The gel strength was mainly affected by the amylose content.

### 3.6. Starch Molecular Structure

The vital molecular structure parameters of SRS are given in Table 1. The molecular weight distributions (M_n_) of rice starch are related to the content of amylose, amylose to amylopectin ratio, and the chain-length distribution of amylopectin. The homogeneous rice varieties showed similar molecular weight distributions. The M_n_ of SRS varied from 1.64 × 10^8^ g/mol to 2.00 × 10^8^ g/mol. SXJ 100 exhibited the highest M_n_ while NJ 46 showed the lowest M_n_. The M_n_ of SRS was lower than WRS while superior to the JRS. Moreover, the polydispersity index (PDI) and z-average radius of gyration (*Rz*) of SRS ranged from 2.16 to 2.58 and 423.1 to 480.4, respectively, which were the highest values of collected rice starch. The higher PDI indicated the different proportions of long chain and short chain in the starch. The molecular density (*ρ*) of soft rice starch varied from 4.09 to 5.71, which was between starch from the waxy rice and japonica rice. The results indicated that the branch of starch from soft rice was less than that of waxy rice. It may be associated with the different rice varieties and the growth environment. NJ 46 and NJ 9108 presented the approximate M_n_ and *ρ*, which illustrated the starch structure was closely related to rice genotypes.

**Figure 3:**
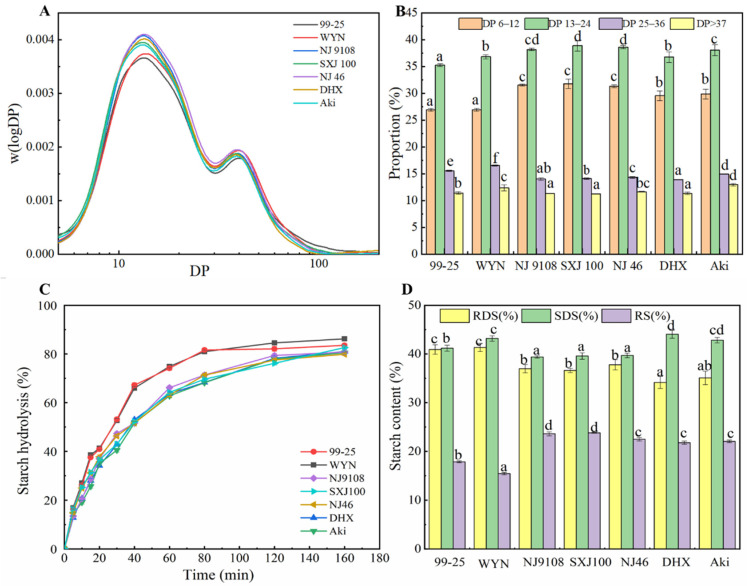
Size exclusion chromatography (SEC) profiles (**A**), chain-length distributions of amylopectin (**B**), digestograms (**C**), and the content of nutritional fractions (**D**) of waxy, soft, and japonica rice starches. Different letters in the same indicators show significant differences (*p* < 0.05).

Figure 3A shows the chain-length distributions of debranched amylopectin from SEC-RID. The chain-length distributions are plotted using logarithmic coordinates. The chain-length distributions are normalized to obtain the maximum values so as to eliminate the influence of the starch concentrations in the different rice varieties. The starch samples exhibited a typical chain-length distribution of amylopectin and presented four peaks and/or shoulders. As per the degree of polymerization (DP), the amylopectin branch chains are divided into four types: A chain, B_1_ chain, B_2_ chain, and B_3_ chain, and the corresponding DP values are 6–12, 13–24, 25–36, and >37, respectively. In Figure 3A, the first peak represents the short amylopectin chains, which contains the A chain and B_1_ chain, while the second peak manifests the medium and long chains of amylopectin, which contains the B_2_ and B_3_ chain. A significant difference was observed in the chain-length distributions among the different rice starches (Table 1). The proportion of each amylopectin category for the rice starch samples is calculated and presented in Figure 3B. The proportions of the A chain, B_1_ chain, B_2_ chain, and B_3_ chain of SRS were 31.32% to 31.78%, 38.19% to 38.87%, 14.01% to 14.32%, and 11.27% to 11.66%, respectively. Compared to starch from waxy rice, the starch from soft rice had the highest proportion of A chain and B_1_ chain, which indicated that amylopectin contained more short chains in the SRS. The fine structure of SRS may lead to its high P_temp_. The stable double helix was formed and passed through into the crystal layer due to the higher proportion of the B_1_ chain. Therefore, soft starch gelatinization needs more energy and higher temperature.

### 3.7. In Vitro Digestion Properties

Based on the starch hydrolysis rate, the nutritional fractions of starch can be classified as rapidly digestible starch (RDS), slowly digestible starch (SDS), and resistant starch (RS), as well as the digestion time corresponds to less than 20 min, 20–120 min, and more than 120 min. The digestograms and nutritional properties among different rice starches are shown in Figure 3C and Figure 3D, respectively. The nutritional fractions (RDS, SDS, and RS) presented a remarkable difference in a variety of rice starches. RDS, SDS, and RS among different rice starches varied from 34.16% (DHX) to 41.34% (WYN), 39.40% (NJ 9108) to 43.20% (WYN), 15.45% (WYN) to 23.81% (SXJ 100), respectively. Compared with starch from soft rice and japonica rice, the WRS presented a higher digestion rate at each time point and exhibited homogeneous digestograms. The higher digestibility of WRS may be ascribed to the large number of amylopectin molecules, which are more easily attacked by amylolytic enzymes. After 120 min, the starch hydrolysis percentage of RS was higher than starch from soft rice and japonica rice. SDS was lower, and RS was higher in SRS than the fractions in WRS and JRS. Amylose content was observed to be distinctly negatively associated with RDS while positively linked with the SDS and RS, and the result was consistent with the report of Chung et al. [35]. The vitro digestion properties are influenced by many factors, including granule morphology, starch–lipid complex, amylose content, amylopectin chain-length distributions, relative crystallinity, and phosphorus content [36].

### 3.8. Correlations between Molecular Structure and Physicochemical Properties of Rice Starch

**Figure 4:**
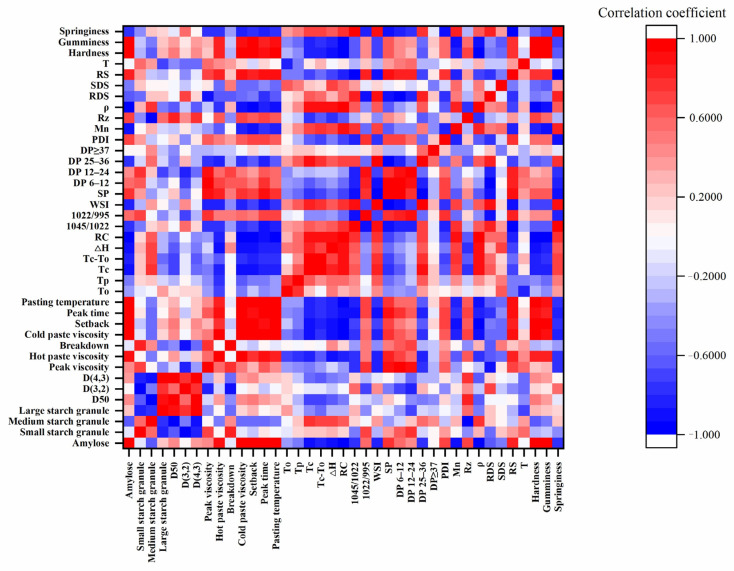
The heat map of correlations between physicochemical properties and molecular structure of waxy, soft, and japonica rice starches.

The relationship between molecular structure and physicochemical properties of rice starch was analyzed in this research. Pearson’s correlation coefficients are exhibited in Figure 4. Amylose content in the rice starch samples was positively correlated with the HPV, CPV, SB, P_time_, P_temp_, hardness, and gumminess but showed no correlation with PV and BD. Kong and Noda et al. also demonstrated a positive relationship between HPV, CPV, and SB with the amylose content of rice starches [7,37]. The results showed that CPV was mainly caused by the repolymerization of amylose molecules during the cooling process [32]. The amylose content was negatively linked with WSI whereas positively correlated with SP. Moreover, the amylose content was negatively related to the ΔH, RC, as well as the ratio of 1047 to 1022 cm^−1^. The amylose could restrain and keep the starch granules swollen, and starches with high RC and the short-range order degree were easier to interact with water [38]. These results were consistent with previous work [39]. In addition, the amylose content was related to molecular weight distributions and chain-length distributions. The amylose content was negatively associated with Mn, *ρ*, and B_2_ chain content and positively related with B_1_ chain content and PDI. These results demonstrated that physicochemical properties and molecular structure were influenced by the amylose content of starch from different rice. 

The PV was positively linked to A and B_1_ chain content while negatively related to B_2_ chain content. The results showed that the short chain of amylopectin influenced the starch granule swelling and was easier to leach out in the starch solution during the heating process [32]. The long chain of amylopectin and the large molecular weight could lead to a higher steric hindrance and inhibit the phenomenon of starch granules swollen. RC and the ratio of 1047 to 1022 cm^−1^ were negatively correlated with A chain content, while positively correlated to B_2_ chain content, M_n_, and *ρ*. The A_1_ chain content was positively relative to SP and negatively relative to WSI. The enrichment of short chains in the starch crystallization zone could reduce the stability of crystallization stacking [40]. The starch granules with a mass of short chains are more likely to swell and reach a higher SP. These results illustrated that the molecular structure had a significant influence on the physicochemical properties of rice starch.

## 4. Conclusions

This study mainly investigated the structure at the molecular level and physicochemical properties of starch from different rice. Compared with starch from waxy rice and japonica rice, starch from soft rice showed different molecular structure and physicochemical properties. The SRS presented low amylose content, which is classified as a very low amylose type. The granule of SRS was polygonal and spherical, and the SRS achieved the highest percentage of small starch granules. The relative crystallinity of SRS varied from 26.15% to 27.55% with a lower short-range ordered structure than WRS. Additionally, the SP and WSI of starch from soft rice were higher than 26.64 g/g and 24.09%, separately. The SRS presented the highest PV and BD among different rice starches, but the values of HPV, CPV, SB, P_time_, and P_temp_ were between starch from waxy rice and japonica rice. Moreover, the Mn and *ρ* of SRS varied from 1.64 × 10^8^ g/mol to 2.00 × 10^8^ g/mol and 4.09 to 5.71, respectively, which was higher than the JRS and lower than WRS. The SRS had the highest proportion of the B_1_ chain. Furthermore, the RS content of SRS was superior to that of WRS and JRS. Correlation analysis proved that the molecular structure and physicochemical properties were influenced by the amylose content of different rice starches. The content of the A_1_ chain and B_3_ chain had an important effect on the physicochemical properties of rice starch. These results provided a theoretical basis for the processing and application of soft rice starch and the breeding of high-quality rice.

## Figures and Tables

**Table 1:** Volume distribution, relative crystallinity, IR ratio, chain-length distributions of amylopectin, and molecular weights of starch granules extracted from waxy, soft, and japonica rice.

	Varieties	99-25	WYN	NJ 9108	SXJ 100	NJ 46	DHX	Aki
	Amylose (%)	0.00 ± 0.00 ^a^	0.00 ± 0.00 ^a^	11.28 ± 0.18 ^c^	10.76 ± 0.34 ^b^	11.85 ± 0.41 ^c^	21.60 ± 0.01 ^e^	18.94 ± 0.19 ^d^
Volume distribution	Small starch granule (<2 μm) (%)	17.54 ± 0.05 ^a^	20.00 ± 0.96 ^bc^	21.55 ± 0.85 ^d^	21.12 ± 0.76 ^cd^	21.23 ± 0.07 ^cd^	16.80 ± 0.00 ^a^	19.37 ± 0.03 ^b^
	Medium starch granule (2–10 μm) (%)	75.73 ± 0.24 ^c^	78.11 ± 0.36 ^e^	73.94 ± 0.12 ^b^	76.78 ± 0.12 ^d^	76.99 ± 0.07 ^d^	70.63 ± 0.05 ^a^	76.32 ± 0.16 ^d^
	Large starch granule (>10 μm) (%)	6.73 ± 0.29 ^c^	1.88 ± 0.60 ^a^	4.00 ± 0.98 ^b^	2.10 ± 0.63 ^a^	1.77 ± 0.00 ^a^	6.94 ± 0.03 ^c^	4.30 ± 0.20 ^b^
	D_50_ (μm)	5.867 ± 0.00 ^d^	5.449 ± 0.02 ^a^	5.854 ± 0.01 ^d^	5.536 ± 0.02 ^b^	5.642 ± 0.03 ^c^	6.125 ± 0.01 ^e^	5.664 ± 0.05 ^c^
	D_(3,2)_ (μm)	3.37 ± 0.01 ^cd^	3.24 ± 0.02 ^bc^	3.02 ± 0.07 ^a^	3.01 ± 0.08 ^a^	3.00 ± 0.00 ^a^	3.47 ± 0.00 ^d^	3.17 ± 0.09 ^ab^
	D_(4,3)_ (μm)	5.67 ± 0.04 ^c^	5.11 ± 0.02 ^a^	5.45 ± 0.08 ^b^	5.15 ± 0.07 ^a^	5.21 ± 0.00 ^a^	5.91 ± 0.01 ^d^	5.41 ± 0.02 ^b^
Crystal structure	Relative crystallinity (%)	30.84	31.15	26.5	27.55	26.15	22.05	22.32
	1047/1022 cm^−1^	0.907 ± 0.017 ^c^	0.892 ± 0.013 ^bc^	0.849 ± 0.000 ^ab^	0.862 ± 0.002 ^abc^	0.842 ± 0.011 ^a^	0.863 ± 0.006 ^abc^	0.843 ± 0.001 ^a^
	1022/955 cm^−1^	1.200 ± 0.049 ^a^	1.259 ± 0.013 ^b^	1.345 ± 0.023 ^c^	1.297 ± 0.007 ^bc^	1.307 ± 0.017 ^bc^	1.270 ± 0.019 ^b^	1.318 ± 0.012 ^bc^
Chain-length distributions of amylopectin	DP 6–12 (%)	26.95 ± 0.25 ^a^	26.93 ± 0.26 ^a^	31.54 ± 0.19 ^c^	31.78 ± 0.87 ^c^	31.32 ± 0.29 ^c^	29.56 ± 0.89 ^b^	29.86 ± 0.89 ^b^
	DP 12–24 (%)	35.26 ± 0.24 ^a^	36.83 ± 0.39 ^b^	38.19 ± 0.24 ^cd^	38.87 ± 0.95 ^d^	38.62 ± 0.30 ^d^	36.77 ± 0.99 ^b^	38.07 ± 0.99 ^c^
	DP 25–36 (%)	15.54 ± 0.12 ^e^	16.54 ± 0.10 ^f^	14.01 ± 0.20 ^ab^	14.09 ± 0.16 ^b^	14.32 ± 0.18 ^c^	13.92 ± 0.03 ^a^	14.92 ± 0.03 ^d^
	DP ≥ 37 (%)	11.42 ± 0.25 ^b^	12.36 ± 0.58 ^c^	11.30 ± 0.03 ^a^	11.27 ± 0.05 ^a^	11.66 ± 0.04 ^bc^	11.35 ± 0.20 ^a^	12.95 ± 0.20 ^d^
Molecular weight	PDI	2.03 ± 0.00 ^b^	1.89 ± 0.01 ^a^	2.58 ± 0.01 ^e^	2.16 ± 0.03 ^c^	2.42 ± 0.09 ^d^	2.22 ± 0.07 ^cd^	2.66 ± 0.01 ^f^
	M_n_ (10^8^ g/mol)	2.29 ± 0.00 ^e^	2.34 ± 0.10 ^e^	1.76 ± 0.11 ^cd^	2.00 ± 0.24 ^d^	1.64 ± 0.18 ^c^	1.50 ± 0.16 ^b^	0.85 ± 0.08 ^a^
	Rz (nm)	413.6 ± 0.14 ^b^	402.75 ± 10.25 ^a^	480.4 ± 1.85 ^e^	423.1. ± 1.37 ^c^	433.2 ± 0.92 ^d^	560.2 ± 4.53 ^f^	423.7 ± 3.07 ^c^
	*ρ* (g/mol/nm^3^)	6.27 ± 0.01 ^g^	6.76 ± 0.18 ^f^	4.09 ± 0.12 ^c^	5.71 ± 0.26 ^e^	4.88 ± 0.08 ^d^	1.98 ± 0.24 ^a^	3.00 ± 0.11 ^b^

Data are means ± standard deviations. Values in the same column followed by different superscripts are significantly different (*p* < 0.05).

**Table 2:** Swelling power, water solubility index, pasting, and thermal properties of starches extracted from waxy, soft, and japonica rice.

	Varieties	99-25	WYN	NJ 9108	SXJ 100	NJ 46	DHX	Aki
Solubility properties	WSI (%)	55.45 ± 0.39 ^d^	67.57 ± 2.12 ^e^	26.72 ± 0.77 ^bc^	25.97 ± 0.94 ^b^	24.09 ± 0.56 ^a^	28.53 ± 0.48 ^c^	29.15 ± 0.26 ^c^
	SP (g/g)	7.63 ± 0.46 ^b^	3.89 ± 0.99 ^a^	27.29 ± 1.20 ^e^	29.30 ± 1.18 ^f^	26.64 ± 0.95 ^e^	24.65 ± 0.56 ^d^	23.80 ± 0.43 ^d^
Pasting properties	Peak viscosity (cP)	2389 ± 9 ^b^	2185 ± 10 ^a^	3614 ± 15 ^e^	3962 ± 12 ^g^	3742 ± 24 ^f^	2760 ± 12 ^c^	3152 ± 20 ^d^
	Hot paste viscosity (cP)	1037 ± 10 ^a^	1054 ± 8 ^a^	1544 ± 3 ^b^	1698 ± 5 ^c^	2006 ± 7 ^d^	2140 ± 7 ^e^	1668 ± 23 ^c^
	Breakdown (cP)	1352 ± 8 ^c^	1130 ± 9 ^b^	2071 ± 1 ^f^	2265 ± 7 ^g^	1736 ± 7 ^e^	620 ± 6 ^a^	1484 ± 17 ^d^
	Cold paste viscosity (cP)	1439 ± 7 ^a^	1434 ± 4 ^a^	2361 ± 5 ^b^	2424 ± 4 ^c^	2818 ± 16 ^d^	3438 ± 8 ^e^	2968 ± 16 ^f^
	Setback (cP)	402 ± 7 ^a^	379 ± 6 ^a^	818 ± 11 ^c^	726 ± 9 ^b^	812 ± 0 ^c^	1299 ± 6 ^d^	1300 ± 7 ^d^
	Peak time (min)	3.36 ± 0.05 ^a^	3.73 ± 0.00 ^b^	5.56 ± 0.05 ^d^	5.13 ± 0.09 ^c^	5.50 ± 0.04 ^d^	6.20 ± 0.10 ^e^	5.56 ± 0.05 ^d^
	Pasting temperature (°C)	67.4 ± 0.64 ^a^	68.6 ± 0.07 ^ab^	70.2 ± 0.14 ^cd^	69.8 ± 0.64 ^bc^	70.2 ± 0.04 ^cd^	71.8 ± 0.17 ^e^	71.4 ± 0.13 ^de^
Thermal properties	To (°C)	57.17 ± 0.21 ^c^	56.88 ± 0.52 ^bc^	57.55 ± 0.08 ^c^	56.00 ± 0.04 ^ab^	55.11 ± 0.22 ^a^	56.05 ± 0.34 ^ab^	56.78 ± 0.86 ^bc^
	Tp (°C)	63.25 ± 0.40 ^c^	65.10 ± 0.37 ^e^	64.37 ± 0.04 ^d^	62.91 ± 0.13 ^bc^	61.75 ± 0.16 ^a^	62.51 ± 0.26 ^b^	63.17 ± 0.28 ^c^
	Tc (°C)	75.63 ± 1.02 ^c^	79.53 ± 1.07 ^d^	72.80 ± 0.10 ^b^	73.55 ± 0.82 ^b^	73.72 ± 0.37 ^b^	69.75 ± 0.45 ^a^	72.00 ± 0.31 ^b^
	Tc-To (°C)	18.46 ± 0.12 ^d^	23.65 ± 0.05 ^e^	15.25 ± 0.08 ^b^	17.55 ± 0.60 ^c^	18.61 ± 0.20 ^d^	13.70 ± 0.16 ^a^	13.70 ± 0.06 ^a^
	ΔH (J/g)	11.80 ± 0.93 ^d^	11.83 ± 0.42 ^d^	10.91 ± 0.41 ^bcd^	11.57 ± 0.12 ^cd^	10.00 ± 0.69 ^ab^	8.88 ± 0.04 ^a^	10.24 ± 0.76 ^abc^
Textural properties and the clarity of starch pastes	Hardness (gf)	118.03 ± 6.44 ^a^	138.92 ± 2.15 ^b^	233.32 ± 5.67 ^c^	235.14 ± 3.57 ^c^	231.29 ± 7.19 ^c^	415.9 ± 7.79 ^d^	362.39 ± 9.64 ^e^
	Gumminess (gf)	91.94 ± 5.03 ^a^	92.31 ± 2.60 ^a^	131.28 ± 6.04 ^b^	178.25 ± 8.7 ^c^	166.46 ± 7.00 ^c^	249.85 ± 7.94 ^d^	249.78 ± 9.03 ^d^
	Springiness	0.83 ± 0.04 ^b^	0.83 ± 0.01 ^b^	0.76 ± 0.03 ^a^	0.78 ± 0.03 ^ab^	0.75 ± 0.02 ^a^	0.78 ± 0.02 ^ab^	0.75 ± 0.01 ^a^
	Transmittance (%)	15.4 ± 0.2 ^bc^	16.9 ± 0.3 ^d^	11.8 ± 0.1 ^a^	18.9 ± 0.2 ^e^	19.4 ± 0.3 ^e^	15.9 ± 0.6 ^c^	14.9 ± 0.1 ^b^

Data are means ± standard deviations. Values in the same column followed by different superscripts are significantly different (*p* < 0.05).

## Data Availability

The datasets generated for this study are available on request to the corresponding author.
